# Short-Term Effects of COVID-19 Lockdown in Italian Children and Adolescents with Type 1 Diabetes Mellitus: The Role of Separation Anxiety

**DOI:** 10.3390/ijerph18115549

**Published:** 2021-05-22

**Authors:** Daniela Di Riso, Simone Bertini, Silvia Spaggiari, Francesca Olivieri, Silvana Zaffani, Lara Comerlati, Marco Marigliano, Claudia Piona, Claudio Maffeis

**Affiliations:** 1 Department of Developmental Psychology and Socialization, University of Padova, 35131 Padova, Italy; daniela.diriso@unipd.it (D.D.R.); simonebertini295@gmail.com (S.B.); 2 Pediatric Diabetes and Metabolic Disorders, Department of Surgical Sciences, Dentistry, Paediatrics and Gynaecology, University of Verona, 37134 Verona, Italy; francesca.olivieri@aovr.veneto.it (F.O.); silvana.zaffani@aovr.veneto.it (S.Z.); lara.comerlati@gmail.com (L.C.); marco.marigliano@aovr.veneto.it (M.M.); claudia.piona@univr.it (C.P.); claudio.maffeis@univr.it (C.M.)

**Keywords:** COVID-19, type 1 diabetes mellitus, children, adolescents, separation anxiety

## Abstract

In March 2020, the Italian Government imposed mandatory home confinement to limit the spread of COVID-19. Few studies assessed the psychophysical impact of COVID-19 on chronically ill children. This study examined these effects on children with Type 1 Diabetes Mellitus (T1D) and their caregivers. Seventy-one patients (7–13 years) with T1D and their caregivers were administered a survey created ad hoc and some standardized questionnaires, assessing psychological well-being and anxiety. Medical data (physical and biochemical characteristics) were recorded before (T_0_, January–February) and after (T_1_, May–June) the lockdown. Paired Student *t*-test, Spearman two-tailed correlations, and a linear regression model were used for statistical analysis. Children at T_1_ showed higher BMI (body mass index), daily total and basal insulin dose, and time spent in therapeutic range, and they showed lower HbA1c (glycated hemoglobin), time spent above the therapeutic range, and standard deviations of the mean glucose values than at T_0_. A total of 32.9% scored in the clinical range for separation anxiety. The increase in separation anxiety was predicted by younger age, female gender, more recent T1D diagnosis, less time spent in therapeutic range at T_1_, and higher perceived fear of COVID-19 infection. In a pandemic context, separation anxiety may be stronger in younger females, with more recent T1D diagnosis and poor metabolic control, thus affecting the parent’s ability to manage diabetes and to support children’s autonomy.

## 1. Introduction

Italy has been the first nation outside of Asia to struggle with the COVID-19 pandemic outbreak, also in terms of confirmed cases and deaths. To contain the transmission of the virus, by 10 March 2020, a national lockdown was imposed by the authorities. Home confinement, severe movement restrictions, closure of non-essential businesses, and schools of every order and degree were established (Phase 1). These rules started to change on the 4 May with a gradual reopening of selected commerce services and softened movement restrictions (Phase 2). Lastly, the Italian Government authorized people to move freely and all activities to restart on 15 June (Phase 3).

Although children seemed to be less prone to be infected by COVID-19, the pandemic might have several psychological consequences on younger individuals due to the unpredictable closure of schools, the interruption of in-person relationships with peers, the breakdown of daily routines, fewer opportunities to discharge their physical energy, and a higher level of distress experienced in prolonged home confinement [1,2]. Preliminary reports on the lockdown’s psychological effects suggest that Italian children and adolescents showed a worsening of sleep quality as well as an increase in emotional symptoms and self-regulation fatigue [3,4]. More specifically, in environmental risk situations such as pandemics, which account for intense worrying for personal and loved ones’ safety, a crucial construct to examine might be separation anxiety [5,6]. It is possible to suppose that a global pandemic such as the COVID-19 outbreak may impact children’s mental health as a traumatic experience does [7]. Some studies reference that separation anxiety is higher in children who have faced a traumatic event in their life and may last for years after it [8]. It could be interesting to observe how children with pediatric chronic diseases could react to a pandemic scenario, in terms of psychological symptoms and management of their medical condition. On one hand, they have to face not only all the stressors related to the pandemic but also all the triggers related to having a chronic condition. On the other hand, it is interesting to explore how the prolonged proximity to their caregivers could impact the care of their medical regimen.

In specific, children with T1D, in normal contexts, seem to have higher prevalence of anxiety and depression when compared to their healthy peers, due to the difficulties imposed by their medical conditions, including the daily basis complex, intrusive treatment regimen, and frequent medical controls [9]. Literature reports a higher prevalence of separation anxiety between children with TD1 compared to healthy children, especially in the youngest. Zaffani et al. hypothesize that parents of these children tend to be oversensitive to their child’s problems and to underestimate their quality of life because of the disease; thus, they tend to be more supportive than necessary, probably eliciting the development of separation anxiety [9]. Furthermore, in T1D, a higher level of separation anxiety is associated with non-adherence to treatment and poor metabolic control [10]. Moreover, parental separation anxiety, worsened by worries about acute diabetes complications or the child’s adequacy to self-monitor their diabetes, was suggested to be detrimental in supporting a healthy pattern of self-management and diabetes control in adolescents with T1D [11].

Few studies have explored the psychological outcomes of T1D pediatric patients in a pandemic outbreak. Children and adolescents with T1D showed a good adjustment to home confinement in terms of healthy habits and metabolic control [12]. Regarding the relationship between psychological well-being and metabolic control, research shows that higher stress was reported by those with higher glycated hemoglobin (HbA1c) levels over the preceding 6 months. Moreover, a bidirectional relationship between stress and glycemic control has been hypothesized [13]. Contrasting data on the glycemic control of adults with diabetes during the pandemic are available. In particular, on the one hand, poor glycemic control, as a consequence of reduced physical activity, availability of healthy food, anti-diabetic medications, and in-person routine follow-up visits, has been reported [14]. On the other hand, an improvement in short-term glycemic control, due to a relevant slowing down of daily routine activities (e.g., diabetes management or timing and preparation of healthy meals), has also been reported [15]. Nevertheless, independently from the impact of lockdown on glycometabolic control, restrictions due to the pandemic seem to have exacerbated the psychological distress by negatively impacting general mental health and diabetes self-management [16].

Therefore, to the best of our knowledge, few studies have explored the physical and psychological well-being in children and adolescents with T1D during the COVID-19 outbreak. It is expected that imposed and prolonged home confinement could have an impact on the behavior of children and adolescents in terms of adherence to treatment, food habits, and physical activity. Parents might be overcontrolling, interfering with children’s diabetes self-management [17].

The aim of this study was to investigate the psychological well-being immediately after the coronavirus lockdown in a cohort of children and adolescents with T1D, and in their parents, and to assess the relationship between the psychological well-being and the glycemic control in this sample. More specifically, firstly, the children’s medical data were compared before and after the lockdown, expecting a worsening in diabetes control parameters due to the discussed possible lockdown’s effects. Secondly, the psychological well-being of mothers and children was explored after the lockdown to examine the pandemic’s expected negative effects on mental health. Moreover, according to the literature, associations between children’s separation anxiety symptoms and glycometabolic variables was expected. Lastly, a multiple regression model was constructed to evidence some possible predictors of children’s separation anxiety symptoms.

## 2. Materials and Methods

### 2.1. Subjects

Seventy-three children and pre-adolescents with T1D attending the Regional Center for Pediatric Diabetes of the University Hospital of Verona, and their respective caregivers (78.9% mothers), were enrolled in the present study. Two out of the 73 patient–caregiver couples did not agree to participate in the research. The inclusion criteria were age between 7 and 13 years, and T1D duration of at least one year. Exclusion criteria were comorbidity with psychiatric and neurological disorders and poor comprehension of the Italian language. No reward was offered for enrollment. The project was approved by the Institutional Ethical Committee of Verona (Prot. n. 29097).

### 2.2. Procedure

A paper-and-pencil survey was administered in person from 18 May to 18 June 2020. Up to 4 May, due to the mandatory interruption of nonessential productive and aid activities, routine visits were replaced by one planned call or video call to monitor both medical and psychological wellness. The study was introduced to parents by the psychotherapist of the ward in agreement with the medical staff, during scheduled visits in the hospital. The administrations were planned in order to not interfere with medical procedures. Parents who agreed to participate signed the informed consent after reading a detailed informative flyer. Each patient and caregiver filled out the questionnaires in a quiet room in the pediatric ward. The survey took about 30 min to complete and consisted of a survey created ad hoc to assess the impact of the COVID-19 outbreak on parents and children, and it also included standardized self-reports assessing the general well-being of the caregivers (General Health Questionnaire, [18]) and the psychological functioning and anxiety symptoms of the children (Strengths and Difficulties Questionnaire, [19]; Spence Children Anxiety Scale, [20,21]).

### 2.3. Measures

#### 2.3.1. Physical Characteristics, Insulin Therapy, and Glucose Monitoring

For each patient who joined the study, diabetes-specific data were recorded twice: at the outpatient visit before the COVID-19 lockdown (T_0_, January–February 2020) and at the outpatient visit at the end of the lockdown (T_1_, 18 May–18 June 2020). Physical characteristics (height, weight, BMI, BMI-SDS), HbA1c, diabetes duration, type of treatment (MDI or CSII), daily insulin doses (total, basal, and prandial dose), and type of glucose-monitoring used (isCGM, rtCGM, or SMBG) were collected from the clinical chart of each subject.

Two-week glucose sensor data were collected twice: before the outpatient visit before the COVID-19 lockdown, and before the outpatient visit at the end of COVID-19 lockdown. Several glucose metrics were derived from the AGP analysis: percentage of time below the range (<70 mg/dL) (%TBR), percentage of time in the target range (70–180 mg/dL) (%TIR), percentage of time above the range (>180 mg/dL) (%TAR), standard deviation of the mean glucose, coefficient of variation (%CV), and Glucose Management Indicator (GMI) [22].

Psychological and psychiatric comorbidities (i.e., anxiety, depression, eating disorders) were screened during the standard outpatient follow-up visits by using standardized tests and questionnaires. The possible diagnosis of diabetic neuropathy and/or other neurological disorders was assessed by evaluating neurological symptoms, based on the diabetic neuropathy symptoms score, and standard clinical tests, as recommended by current International Society for Pediatric and Adolescent Diabetes (ISPAD) guidelines [23]. As to the ad hoc survey and the standardized questionnaires, they were completed during the outpatient visit at the end of the lockdown (T_1_, 18 May–18 June 2020).

#### 2.3.2. Psychological Factors and Sociodemographic Characteristics: The Ad Hoc Survey

An ad hoc survey was created for the present study, with one part for caregivers and another one for children. Parents had to report socio-demographic characteristics including gender, age, education, and working conditions during the lockdown (10 March-4 May) and after the lockdown (after 4 May). As to the educational level, caregivers were asked about the maximum number of years of study (5 for primary school, 8 for middle school, 13 for secondary school, 16–20 for graduation). Regarding the working conditions, the possible answers were the following: “I worked outside”, “I had to stop my work and I’m in layoffs”, “I had to stop my work and I’m unemployed”, “I still worked at home (i.e., housewife)”, “I worked in smart-working form”. They were asked about their children’s T1D management (e.g., children’s degree of autonomy in handling diabetes medications, on a 5-point Likert scale from 1 “completely parents dependent” to 5 “totally independent”), family’s communication about T1D (on a 5-point Likert scale from 1 “totally absent” to 5 “excellent”), and time of parent–child relationship (hours a day) before and during the COVID-19 pandemic. Moreover, we assessed how worried they were about their children’s contagion on a 3-point Likert scale, from 1 “not at all”, 2 “quite worried”, to 3 “a lot” (e.g., “Do you think that Covid-19 may be more dangerous for your child due to his/her medical condition (T1D)?”), and about the resumption of their children’s activities on a 5-point Likert scale from 1 “not at all” to 5 “extremely worried”. Specific items explored the perceived change in parents’ and children’s physical and psychological well-being compared to the period before the COVID-19 pandemic (“If you think about your psychological well-being before the COVID-19 pandemic, how would you evaluate it compared to now? It was worse/the same/better than now”; “If you think about your child’s psychological well-being before the COVID-19 pandemic, how would you evaluate it compared to now? It was worse/the same/better than now”). Higher rates in these last items indicate worse psychological well-being at the time of the compilation compared with the period before the pandemic.

The same questions were administered to children. They were also asked about the number of siblings and the frequency of contact with peers during one week (on a 3-point scale: 1 “at most 2 times”, 2 “from 2 to 4 times”, 3 “more than 4 times”) and how worried they were about getting infected by COVID-19.

#### 2.3.3. Psychological Factors: The Standardized Questionnaires

Caregivers’ well-being was assessed using the General Health Questionnaire (GHQ-12) [18,24]. Through 12 items rated on a 4-point Likert scale, the GHQ-12 assesses the presence of minor psychological disorders in primary care settings. GHQ-12 total scores were distinguished into three ranges: no presence of difficulties, presence of minor difficulties, and presence of important difficulties that could need professional intervention. In this study, Cronbach’s α was α(GHQ-12) = 0.703.

Psychological functioning in children was assessed using the Strengths and Difficulties Questionnaire (SDQ) [19,25]. The questionnaire is a validated behavioral screening composed of 25 items rated on a 3-point Likert scale that assess emotional symptoms, conduct problems, hyperactivity and inattention, peer problems, and prosocial behaviors. By adding the first four scales, a total difficulties score can be calculated. In this study, Cronbach’s α for the total score (TDS), the internalizing symptoms scale (INT), the externalizing symptoms scale (EXT), and the prosocial behaviors scale (PROS) were, respectively, α(TDS) = 0.592, α (INT) = 0.614, α(EXT) = 0.606, and α(PROS) = 0.731.

Children’s anxiety symptoms were assessed using the Spence Children Anxiety Scale (SCAS) [20,21,26]. The questionnaire is composed of 44 items to which children need to answer by marking how often they experience the described situations on a 4-point Likert scale. Of all items, 38 are about specific anxiety symptoms, while six are filler items. The questionnaire comprises 6 dimensions: panic and agoraphobia (PA), separation anxiety (SAD), fears of physical injury (PHY), social phobia (SOC), obsessive-compulsive problems (OCD), and generalized anxiety/overanxious symptoms (GAD). Adding all the scores, a total score (TOT) can be calculated. In this study, Cronbach’s α for each scale was α(PA) = 0.718, α(SAD) = 0.635, α(PHY) = 0.407, α(SOC) = 0.630, α(OCD) = 0.548, α(GAD) = 0.699, and α(TOT) = 0.835.

### 2.4. Data Analysis

Percentages of TDS-SDQ, SCAS total, SCAS subscales, and GHQ-12 clinical levels were provided according to normative data.

Non-clinical and clinical levels of the children’s and mothers’ psychological variables (TDS-SDQ, SCAS total, and SCAS subscales) and patients’ characteristics were reported as mean and standard deviation (SD) or as percentages. The Kolmogorov–Smirnov test was used to assess the normal distribution of variables. Skewed variables were transformed (natural log-transformed, if and as needed) to correct for non-Gaussian distribution unless deviations from the Gaussian distribution could not be corrected by transformation. Paired Student *t*-tests were carried out to assess metabolic and anthropometric differences between T_0_ and T_1_. A multivariate analysis of variance (MANOVA), with age and gender as independent variables, was used to analyze the age and gender differences in children’s emotional well-being.

Spearman two-tailed correlations were performed between SCAS-SAD and children’s age, children and mother’s psychological functioning (GHQ-12), diabetes-specific variables (%TIR, %TAR, diabetes duration), and selected psychosocial variables from the survey (children autonomy in T1D management, children perceived fear of COVID-19 infection, frequency of contacts with peers, time of mother–child relationship).

A multiple linear regression model with SCAS-SAD as the dependent variable and, as independent variables, age, gender, diabetes duration, %TIR at T_1_ (%TIR_T1_), children autonomy in T1D management, and children perceived fear of COVID-19 infection was performed to assess the relationship between separation anxiety symptoms, diabetes-specific variables, and demographic and psychosocial variables. The stepwise method was used.

A *p*-value < 0.05 was considered to be statistically significant. Statistical analysis was performed using the SPSS v22.0 software package (SPSS Inc., Chicago, IL, USA).

## 3. Results

Seventy-one patients were recruited (males 53.4%), mean age 11 (2.26) years (age-range: 7–13 years). Regarding insulin therapy, 52.1% of patients were on multiple daily injections (MDI) and 47.9% on continuous subcutaneous insulin infusion (CSII). Intermittently scanned continuous glucose monitoring (isCGM) was used by 32.4% of patients, 59.2% of them used real-time continuous glucose monitoring (rtCGM), and 8.5% used self-monitoring of blood glucose (SMBG). Seventy-one caregivers (78.9% mothers) took part in the study. In Table 1 we reported demographics and descriptive information of parents and their children. In Table 2, COVID-19-related questions from the ad hoc survey are listed.

### 3.1. Comparison of Physical Characteristics, Insulin Therapy Dose, and Glycometabolic Variables before and after the Lockdown

Children at T_1_ showed significantly higher body weight and height, BMI, daily total and basal insulin dose, and %TIR than at T_0_. Furthermore, significantly lower HbA1c, %TAR, and standard deviation of the mean glucose values were found in T_1_ than at T_0_. No significant differences were detected in BMI-SDS, daily prandial insulin dose, daily total insulin, %TBR, %CV, and GMI values (Table 3).

### 3.2. Psychological Well-Being in Mothers and Children Immediately after the Lockdown

Our study showed that 14.9% of children scored above the clinical cut-off for SDQ-TDS and 6.7% for SCAS total. As for SCAS subscales, 32.9% for SCAS-SAD, 21.2% for SCAS-PHY, 20.6% for SCAS-GAD, 16.7% for SCAS-SOC, 7.8% for SCAS-PA, and 3.1% for SCAS-OCD accounted for the clinical range.

A multivariate analysis of variance (MANOVA), with age and gender as independent variables, was used to analyze age and gender differences in children’s emotional well-being. The sample was divided into two groups on the basis of age: 7 to 10 years old (primary school), and 11 to 14 years old (secondary school). Children at primary school had significantly higher separation anxiety scores (F = 8.376, df = 1, *p* = 0.006) (M = 6.84, DS = 3.78) when compared with secondary school children (M = 4.08, DS = 2.68). As to the social anxiety, a significant difference (F = 9.918, df = 1, *p* = 0.003) was found between children at primary and secondary school: those last had higher scores (M = 6.20, DS = 3.24) when compared with younger children (M = 3.78, DS = 2.82).

Moreover, gender differences need to be taken into account. As to the variables “physical injuries”, “social anxiety”, and “SCAS total score”, females of the clinical samples scored significantly higher (F = 4.739, df = 1, *p* = 0.034; F = 6.367, df = 1, *p* = 0.015; F = 6.475, df = 1, *p* = 0.014) than males (physical injuries: M = 2.95, DS = 2.01 for females, M = 1.93, DS = 1.96 for males; social anxiety M = 6.37, DS = 3.11 for females, M = 4.53, DS = 3.23 for males; SCAS total score: M = 26.79, DS = 10.49 for females, M = 19.36, DS = 10.35 for males). Regarding the interaction between age and gender, no significance was found.

Although the present study did not include retrospective children’s psychological functioning, 67.6% of caregivers reported that their children’s psychological well-being was unvaried or ameliorated immediately after the lockdown. Following, 76.5% of mothers indicated that T1D patients’ physical well-being was stable or improved.

Regarding general health measured by GHQ-12, 47.1% reported minor difficulties experienced immediately after the lockdown, 26.5% reported no difficulties, and 26.4% reported important impairment. As to the ad hoc survey, 50% of mothers reported that their psychological well-being was unvaried or ameliorated immediately after the lockdown compared to the period before the COVID-19 pandemic. Following, 63.8% of mothers indicated that their physical well-being was stable or improved.

### 3.3. Associations between Separation Anxiety Symptoms and Glycometabolic Variables

SCAS-SAD showed positive association with %TAR_T1_ (*r* = 0.327, *p* = 0.016), and children perceived fear of COVID-19 infection (*r* = 0.296, *p* = 0.006). SCAS-SAD was negatively associated with the children’s age (*r* = −0.576, *p* < 0.001), diabetes duration (*r* = −0.292, *p* = 0.016), %TIR_T1_ (*r* = −0.276, *p* = 0.018), children’s autonomy in T1D management (*r* = −0.359, *p* = 0.004), and frequency of contacts with peers (*r* = −0.288, *p* = 0.047). No significant correlations were found between SCAS-SAD and mothers’ GHQ-12 and time of mother–child relationship (data not reported).

The multiple regression model indicated that the increase in children’s separation anxiety symptoms was predicted by age (younger children), gender (female), diabetes duration (more recent T1D diagnosis), %TIR_T1_ (less time spent in target range), and an increased children’s perceived fear of COVID-19 infection (Table 4). Interestingly, the children’s autonomy in T1D management was not a significant predictor of the increase in separation anxiety symptoms. In addition, testing other models, including other diabetic-specific variables (e.g., %TAR) or pandemic-related variables (e.g., frequency of contacts with peers), did not improve the presented model (data not reported).

## 4. Discussion

The current study focused on the psychological and physical well-being of children with T1D during the COVID-19 outbreak period. The psychological functioning was assessed immediately after the mandatory home confinement (from 18 May–18 June 2020), during in-person follow-up visits. The diabetes-specific functioning was evaluated in the same period and compared with metabolic data collected in January/February 2020.

Concerning psychological functioning, most of the children with diabetes reported a normative level of general and anxiety-related symptoms. Even though we did not dispose of psychological measures administered in a pre-COVID period, for most of the children, parents reported that the general psychological well-being was unchanged.

Consistent with the literature, it was found that separation anxiety was more frequent in younger children and females [5], and that females were generally more anxious than males [28].

More than 30% of T1D patients displayed separation anxiety symptoms at a clinical level. Epidemiological studies estimated that the prevalence for clinical anxiety symptoms is from 3% to 5% in children within the same age range [5]. Although future studies need to be carried out to explore the nature of this symptomatology, evidence suggested that T1D children experienced a high level of separation anxiety symptoms immediately after the lockdown.

The present study showed that higher levels of separation anxiety were associated with the younger age and female gender, in agreement with the data reported in epidemiological studies with not-referred samples [5]. Some authors explain the gender difference considering that parents tend to be more tolerant to their daughter’s separation anxiety symptoms than to their son’s [5]. Moreover, it might be considered that anxiety (in general) is more frequent between females compared to males [28]. Future studies need to better explore these possible explanations. Separation anxiety symptoms were associated with a lower duration of diabetes. Presumably children with lower expertise and autonomy in disease management, due to a more recent diagnosis, might be more concerned about being separated from parents, who could be in charge of diabetes medications or complications. In this study, children’s autonomy in diabetes management was not significantly associated with separation anxiety. However, this finding may be due to the strong correlation between autonomy and children’s age: younger children, who are physiologically less autonomous, showed more separation anxiety than the older ones. Autonomy and self-management remain integral parts of every therapeutic education program and must be regularly assessed by specific monitoring sheets, measured and encouraged, according to national and international guidelines [29].

In addition, separation anxiety was associated with the fear of being infected by COVID-19. In emergency contexts, as it is the case with the coronavirus pandemic, in which personal and loved ones’ safety might be at risk, separation anxiety might be exacerbated [6].

Lastly, separation anxiety was associated with glucose metabolic control. In particular, children and adolescents with a higher %TIR and lower HbA1c had milder symptoms of separation anxiety, even though we detected in children and adolescents with T1D, after the lockdown, higher clinical anxiety symptom scores than those reported in the reference general population [10]. Nevertheless, the behavior changes induced by lockdown exposure, i.e., reduction in routine activities and more attention on glucose profile and insulin therapy by the patients and caregivers, had beneficial effects on T1D control in our sample. In fact, after lockdown, children and adolescents showed lower levels of HbA1c and better glycemic metrics from the data of isCGM and rtCGM (improved %TIR, reduced %TAR, and standard deviation of the mean glucose) than before lockdown. These glycometabolic results are in agreement with Fernández et al., who reported a better %TIR measured by isCGM in the 14 days after the lockdown than the 14 days before, in a sample of adults with T1D [30]. Moreover, other samples of children and adults with T1D, who measured their glucose profiles by CGM before and during COVID-19 lockdown, showed an improvement or no changes in their glycemic control [15,31,32]. A potential explanation of this finding may be the fear, of parents and adolescents, that T1D could worsen the outcomes of COVID-19 infection, leading them to pay more attention to diabetes management [33,34]. These glycometabolic results are partially confirmed in our sample by parents who evaluated the physical well-being of their children as stable, comparing the period immediately after the lockdown with a pre-COVID scenario.

Results of the present study should be interpreted in light of some limitations. First, the current study is cross-sectional for the collection of psychological functioning, and the paper is lacking a baseline of children’s symptoms before the lockdown. Secondly, the cause/effect relationship between the variables was not assessed. Third, no data on physical activity and nutritional habits were considered, which are two factors influencing glycemic control. Then, children’s separation anxiety could also be associated with other variables not considered in the present study (e.g., maternal anxiety). Moreover, Cronbach’s alpha coefficients for some of the standardized questionnaires were quite low. Regardless, some studies report low Cronbach’s alpha for some SDQ subscales [35].

This study also has strengths: the sample characteristics, which include data on children and parents; the sample size, which is reasonably adequate considering the narrow age range of children and adolescents recruited; and the use of validated questionnaires, with Italian normative data. Moreover, the data collection scheduled after the lockdown period could highlight the short- and long-term psychological effects of restrictive maneuvers, to organize adequate medical and psychological support programs.

In conclusion, the results of this study suggest that, in a pandemic context, separation anxiety may be experienced in particular by younger children and by those with poor metabolic control. In these cases, children tend to perceive themselves as more vulnerable and to heighten attachment behavior and requests of proximity and protection. Likewise, their separation anxiety, adding to other parental concerns and fears, probably affects the parents’ ability to support their children’s development of autonomy in self-care and self-reliance [36].

Future investigation on the influences of separation anxiety on autonomy and metabolic control in parents and children is recommended, as well as on the contribution of autonomy development to diabetes self-management and quality of life. Furthermore, future lines of research should examine other anxiety dimensions (e.g., obsessive-compulsive disorder), not considered in the present study, in children with diabetes, or put a focus on parents’ separation anxiety. It would also be interesting to develop a longitudinal study, assessing the psychological well-being of families of children with diabetes one year later.

Moreover, it would be important to further examine the psychological impact of the COVID-19 pandemic on chronically ill children and their parents, in order to develop psychosocial support programs for this population. Finding connections between the psychological and physical variables in children with T1D is also relevant, and future studies should examine in depth the link between these to improve caring protocols.

## 5. Conclusions

The current study focused on the psychological and physical well-being of T1D during the COVID-19 outbreak period. Concerning psychological functioning, most of the children with diabetes reported a normative level of general and anxiety-related symptoms. More than 30% of T1D patients displayed separation anxiety symptoms at a clinical level. The increase in those symptoms was predicted by younger age, female gender, more recent T1D diagnosis, less time spent in therapeutic range at T_1_, and higher perceived fear of COVID-19 infection.

## Figures and Tables

**Table 1:** Demographics and descriptive information of parents and children.

Parents (78.9% Mothers)	Mean (SD)	
Age (years) (minimum–maximum)	43.1 (6.2) (27–54)	
Educational level (years)	12.6 (3.5)	
Number of family members (3/4/more)	20%/60%/20%	
Gross income categories	10.6%/21.3%/68.1%	
Work (% for each answer)	During the lockdown (between 10 March and 4 May)	After the lockdown (after 4 May)
“I worked outside”	24.60%	53.00%
“I had to stop my work and I’m in layoffs”	13.00%	7.60%
“I had to stop my work and I’m unemployed”	17.40%	4.50%
“I still worked at home (i.e., housewife)”	26.10%	25.80%
“I worked in smart-working form”	18.90%	9.10%
**Children and Adolescents**	**Mean (SD)**	
Age (years)(Male/Female) (minimum–maximum)	10.8(2.3)/11.3(2.2) (7–14)	
Gender (%) (Male/Female)	39(54.9)/32(45.1)	
Pubertal stage	33/35/3	
Age at the T1D onset	4.86 (2.8)	
Diabetes duration (years)	5.69 (2.96)	
MDI/CSII (*n*, %)	37(52.1)/34(47.9)	
isCGM/rtCGM/SMBG (*n*, %)	23(32.4)/42(59.2)/6(8.5)	
Number of siblings	1.00 (0.74)	
Frequency of contacts with peers (during the week)	2.22 (0.77)	

All results are reported as mean (SD), or with the percentage, where specified. Gross income categories are expressed with income <15,000€, between 15,000€ and 26,000€, and ≥26,000€, respectively. The pubertal stage was assessed according to Tanner criteria [27]. Subjects were categorized in prepubertal (Tanner stage 1), pubertal (Tanner stage 2–4), and post pubertal (Tanner stage 5). (MDI, multiple daily injections; CSII, continuous subcutaneous insulin infusion; isCGM, intermittently scanned continuous glucose monitoring; rtCGM, real-time continuous glucose monitoring; SMBG, self-monitoring of blood glucose).

**Table 2:** COVID-19-related questions from the ad hoc survey for parents and children (valid percentages are reported).

Parents (78.9% Mothers)	Percentages/Mean (SD)	
Caregivers’ concerns about the resumptions of children’s activities		
1, “not at all”	29%	
2, “little worried”	23.30%	
3, “worried”	24.60%	
4, “very worried”	14.50%	
5, “extremely worried”	8.70%	
Caregivers’ concerns about their children’s contagion		
Not at all	29.00%	
Quite worried	56.50%	
A lot	14.50%	
	**Before the COVID-19 Pandemic**	**During the COVID-19 Pandemic**
Children’s Autonomy in T1D management		
1, “completely parents dependent”	4.30%	4.30%
2, “quite parents dependent”	18.8%	21.4%
3, “halfway”	43.5%	40.0%
4, “quite independent”	23.2%	24.3%
5, “totally independent”	10.1%	10.0%
Family Communication about T1D		
1, “totally absent”	0%	0%
2, “poor”	10.10%	4.30%
3, “halfway”	11.6%	17.4%
4, “good”	52.2%	52.2%
5, “excellent”	26.1%	26.1%
Time of parent-child relationship (hours a day)	9.03 (5.44)	16.27 (7.24)
**Children and Adolescents**	**Percentages**	
Children’s perceived fear of COVID-19 infection		
Not at all	45.80%	
Quite worried	45.80%	
A lot	8.30%	

All results are reported as mean (SD), or with the percentage, where specified. T1D, type 1 diabetes.

**Table 3:** Physical characteristics, insulin therapy dose, and glycometabolic variables at the outpatient visit before the COVID-19 lockdown (T_0_) and at the outpatient visit at the end of the lockdown (T_1_).

Variables	T_0_	T_1_	t	*p*
	Mean (SD)	Mean (SD)		
Weight (kg)	40.4 (12.0)	42.4 (12.8)	−8.819	<0.001
Height (cm)	144.5 (14.3)	146.8 (14.3)	−12.393	<0.001
BMI (kg/m^2^)	18.9 (2.8)	19.2 (3.1)	−2.813	<0.05
BMI-SDS	0.72 (0.82)	0.73 (0.87)	0.144	0.888
Total insulin dose (IU/die)	32.3 (16.8)	35.5 (16.4)	−3.086	<0.05
Basal insulin dose (IU/die)	16.7 (9.1)	19.0 (11.2)	−1.965	<0.05
Prandial insulin dose (IU/die)	15.6 (9.6)	17.4 (10.4)	1.713	0.065
Total Insulin (IU/kg/d)	0.77 (0.25)	0.81 (0.23)	−2.218	0.062
HbA1c (%)	7.7 (0.7)	7.5 (0.8)	5.2	<0.001
%TBR	3.3 (2.5)	3.1 (3.0)	0.406	0.546
%TIR	54.3 (13.5)	57.0 (15.2)	−2.983	<0.05
%TAR	42.8 (14.5)	40.2 (14.5)	2.54	<0.05
Standard deviation of the mean glucose	66.5 (14.3)	64.0 (16.1)	2.521	<0.05
%CV	37.5 (4.5)	36.7 (4.6)	1.518	0.058
GMI	7.5 (0.6)	7.4 (0.7)	2.261	0.078

Data are expressed as mean and standard deviation (SD) and paired *t*-test *p*-value. (BMI, body mass index; BMI-SDS, standardized body mass index; %TBR, percentage of time below the range (<70 mg/dL); %TIR, percentage of time in target range (70–180 mg/dL); %TAR, percentage of time above the range (>180 mg/dL); %CV, coefficient of variation; and GMI, Glucose Management Indicator).

**Table 4:** Multiple regression of children’s separation anxiety (SCAS-SAD), (%TIR_T1_, percentage of time in target range (70–180 mg/dL) at T_1_).

	Children’s Separation Anxiety (SCAS-SAD)			
	B (95% CI)	Std. β	*t*	*p*
Intercept	14.069 (9.51, 19.69)		5.787	<0.05
Age (years)	−0.549 (−0.977, −0.093)	−0.344	−2.726	<0.05
Gender (2 = F)	2.016 (0.466, 3.350)	0.278	2.646	<0.05
Diabetes duration (years)	−0.338 (−0.616, −0.081)	−0.274	−2.324	<0.05
Children’s Autonomy in T1D management	−0.113 (−1.013, 0.899)	−0.03	−0.245	0.808
Children’s perceived fear of COVID-19 infection	1.194 (−0.106, 2.511)	0.213	2.095	<0.05
%TIR_T1_	−0.098 (−0.165, −0.047)	−0.411	−3.729	<0.05
Model fit	F(6.50) =9.34			
	*p* < 0.001			
Adj. R^2^	0.5			

Multiple linear regression model of children’s separation anxiety. *B*, unstandardized beta; *std. β*, standardized beta; *CI*, confidence intervals; Adj. R^2^, adjusted R^2^; T1D, type 1 diabetes.

## Data Availability

The data presented in this study are available on request from the corresponding author. The data are not publicly available due to participants’ privacy.
